# A new species of *Odaginiceps* Fiers, 1995 (Copepoda, Harpacticoida, Tetragonicipitidae) from the Mediterranean coast of Turkey

**DOI:** 10.3897/zookeys.53.389

**Published:** 2010-08-27

**Authors:** Suphan Karaytug, Serdar Sak, Alp Alper

**Affiliations:** 1 Mersin University, Faculty of Arts and Science, Department of Biology, 33343, Mersin, Turkey; 2 Balıkesir University, Faculty of Arts and Science, Department of Biology, 10145, Bal?kesir, Turkey

**Keywords:** Harpacticoida, Tetragonicipitidae, Odaginiceps, taxonomy, new species

## Abstract

Male and female of Odaginiceps korykosensis **sp. n.** (Copepoda, Harpacticoida, Tetragonicipitidae), collected in the intertidal zone of Kızkalesi beach along the Mediterranean coast of Turkey (Mersin Province), are described. The new species is the fifth member of the genus and can easily be distinguished from the other species by the presence of four setae/spines on the second endopodal segment of P4 and by the structure of the caudal rami. Previously, representatives of the genus Odaginiceps have been reported from Gulf of Mexico, off Bermuda and Kenya. Odaginiceps korykosensis **sp. n.** is the first record of the genus in the Mediterranean Sea.

## Introduction

The genus Odaginiceps Fiers, 1995 is one of the 12 genera currently recognized in the family Tetragonicipitidae and now comprises four species (Wells 2007). The genus was first created by Fiers (1995) in one of his comprehensive and excellent papers on the Tetragonicipitidae from the Yucatecan continental shelf (Mexico). In this paper, Fiers (1995) described Odaginiceps clarkae Fiers, 1995 from the West Central Atlantic (Quintana Roo State, Nichupte Lagoon, Cancun, Mexico) and designated it as the type species of the genus. In the same paper Fiers (1995) also described Odaginiceps xamaneki Fiers, 1995 from the Western Central Atlantic (western region of Yucatecan continental shelf, Mexico). The third species (Odaginiceps elegantissima Fiers, 1995) was also described and allocated to the genus by Fiers (1995) after reexamining the material previously identified by Coull (1970) as Diagoniceps laevis Willey, 1930 from Castle Harbour (Bermuda). Fiers and De Troch (2000) later described the fourth species, Odaginiceps immanis Fiers and De Troch, 2000, from the Indo-Pacific (Gazi Bay, Kenya). No other report of Odaginiceps has appeared in the literature since then. But the intensive investigation (carried out between 2000–2010) of over 500 phytal and interstitial harpacticoid samples taken from nearly 200 different stations along the mediolittoral zone of rocky shores and sandy beaches of almost all Turkish coasts (unpublished data) revealed a new species of Odaginiceps which was found only in a single locality. Both sexes of this new species were described in detail below.

## Material and methods

Samples were collected using the Karaman-Chappuis method (Delamare Deboutteville 1953) from the type locality on three different dates (Table 1) but only one male and one female were obtained at the first sampling (April 09, 2007). Prior to dissection, the habitus was drawn from whole specimens temporarily mounted in lactophenol. Specimens were dissected in lactic acid and the dissected parts were mounted in lactophenol mounting medium. Broken glass fibres were added to prevent the animal and appendages from being compressed by the coverslip and to facilitate rotation and manipulation, allowing observation from all angles. Preparations were subsequently sealed with Entellan® (Merck). All drawings were prepared using a camera lucida on Olympus BX-51 differential interference contrast microscope. Total body length was measured from the anterior margin of the rostrum to the posterior margin of the caudal rami. Measurements were made with an ocular micrometer. Scale bars in illustrations are in µm. The descriptive terminology is adopted from Huys et al. (1996). Abbreviations used in the text are: ae, aesthetasc; P1–P6, for swimming legs 1–6; exp (enp)-1 (-2, -3) to denote the proximal (middle, distal) segment of a ramus. Material was deposited in the Mersin University Zoology Museum (MUZM) at Mersin, Turkey. Physical and chemical parameters of the interstitial water in the sampling pit are summarized in Table 1. Parameters were measured with an YSI 85 Handheld Dissolved Oxygen and Conductivity Instrument (YSI Inc.), with the exception of the pH which was measured with an Orion 3-star (Thermo Fisher Scientific Inc.) Portable pH Meter.

**Table 1:** Some physical and chemical parameters of interstitial water on different sampling dates at the type locality.

Date	09 April 2007	27 July 2007	26 November 2007
pH	8.16	7.29	8.14
Temperature (°C)	18.3	29.8	19.1
Conductivity (ms)	45.7	57.1	54.0
Salinity (ppt)	34.5	37.8	35.5
Oxygen (mg/L)	2.78	1.11	1.71

## Results

Order Harpacticoida Sars, 1903

Family Tetragonicipitidae Lang, 1944

Genus Odaginiceps Fiers, 1995

### Odaginiceps korykosensis sp. n.

urn:lsid:zoobank.org:act:8EF0600A-D85B-4032-9661-26A514C0ADDD

Figs 1 6 

#### Type locality.

Turkey, Mediterranean coast, Mersin Province; intertidal zone of Kızkalesi beach (36°27.473'N; 34°08.647'E). The type locality is fine sand beach.

#### Material examined:

Holotype ♀ dissected on seven slides. Allotype ♂ dissected on six slides. Legs. S. Karaytuğ, S. Sak, A. Alper and S. Sönmez.

#### Description.

**Figure 1.**
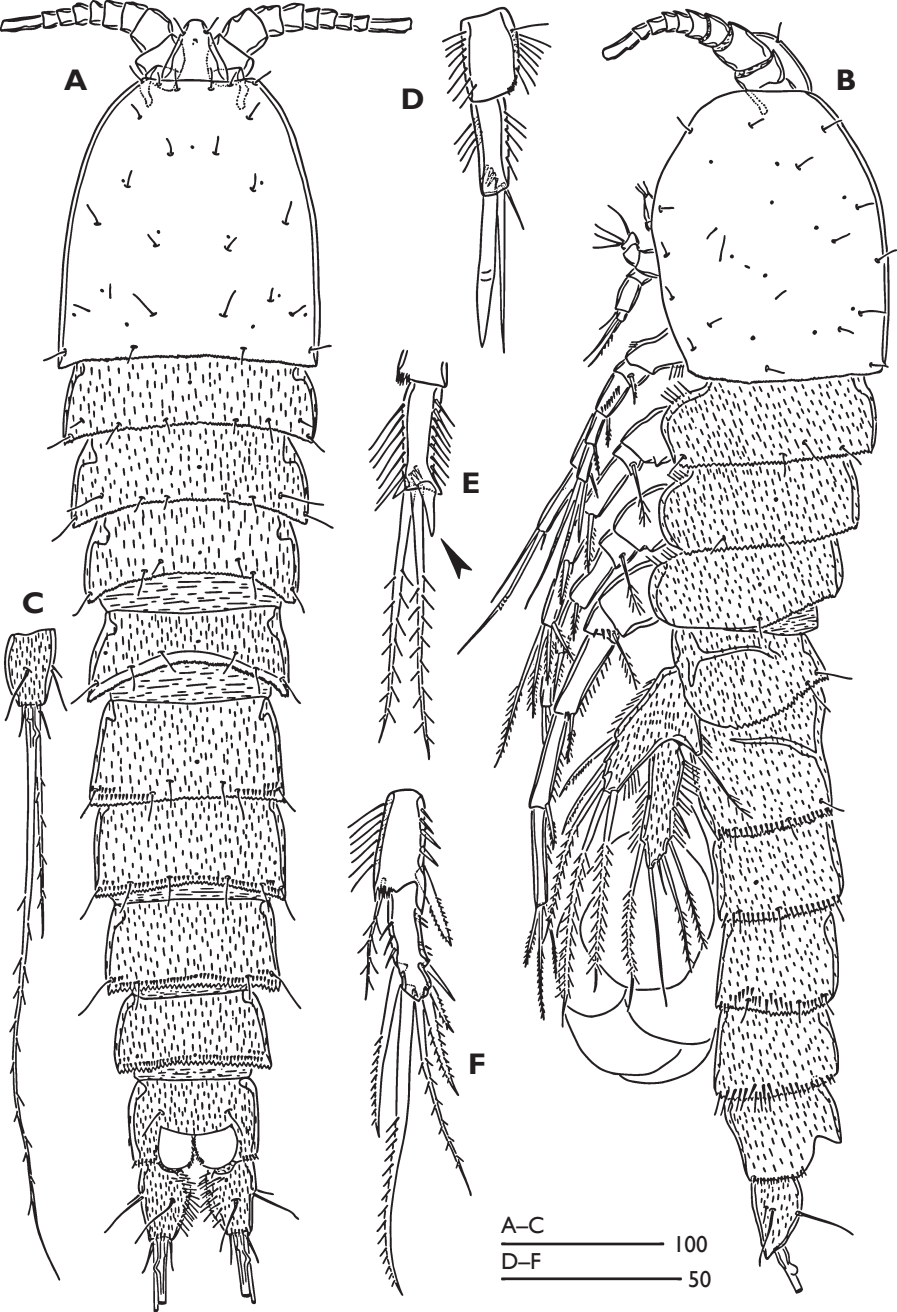
Odaginiceps korykosensis sp. n. Female. **A** habitus, dorsal **B** habitus, lateral **C** right caudal ramus with terminal complement, dorsal. Male. **D** P2 endopod, anterior **E** P3 terminal endopod segment, anterior **F** P4 endopod, anterior.

Female (Fig. 1A, B).Total body length 770 µm, with largest width measured at cephalothorax. Integument of cephalic shield smooth, of all other somites ornamented with irregular pattern of hardly visible spinules. Body surface with sensilla pattern as figured. Posterior margin of the body somites with serrate hyaline frills.

Rostrum (Fig. 1A) large, widest at base, extending halfway along second antennular segment; with two delicate sensillae and a mid-dorsal pore.

**Figure 2.**
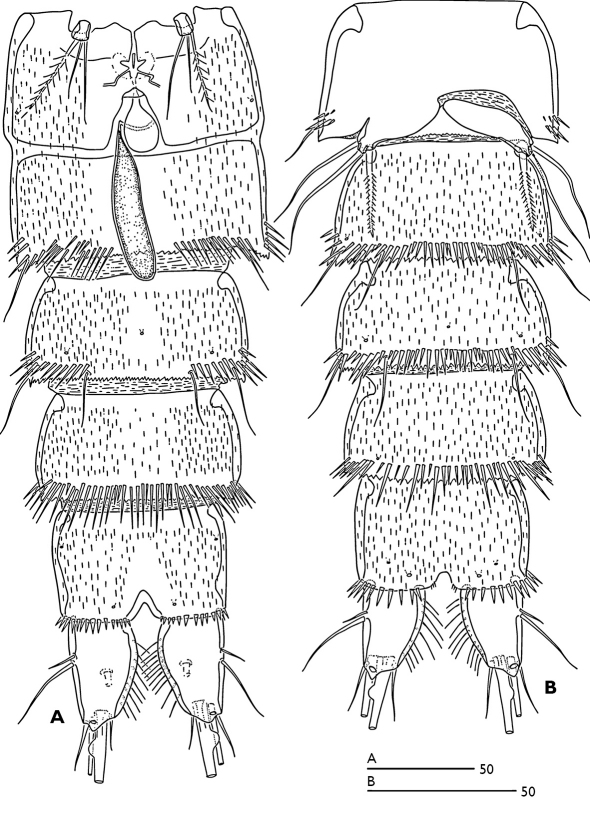
Odaginiceps korykosensis sp. n. **A** female, abdomen, ventral **B** male, abdomen, ventral.

Urosome (Figs 1A, B) 5-segmented, comprising P5-bearing somite, genital double somite and three free abdominal somites. Genital double-somite longer than wide; with transverse surface ridge dorsally and laterally (Fig. 1B) extending ventrally (Figs 1A; 2A), indicating original segmentation. Genital field (Fig. 2A) with small copulatory pore located in median triangular depression. A spermatophore attached to the copulatory pore. First and second somite of genital double-somite and second and third abdominal somites with continuous spinules near distal margin dorsally (spinules of first somite of genital double-somite interrupted midway) (Fig. 1A); genital double somite and second abdominal somite with spinular row near distal margin ventrally, interrupted midway; third abdominal somite with continuous spinular row near distal margin ventrally (Fig. 2A). Anal somite (Fig. 1A) with distal spinular row extending dorsally to the either side of anal operculum; operculum smooth, slightly convex.

**Figure 3.**
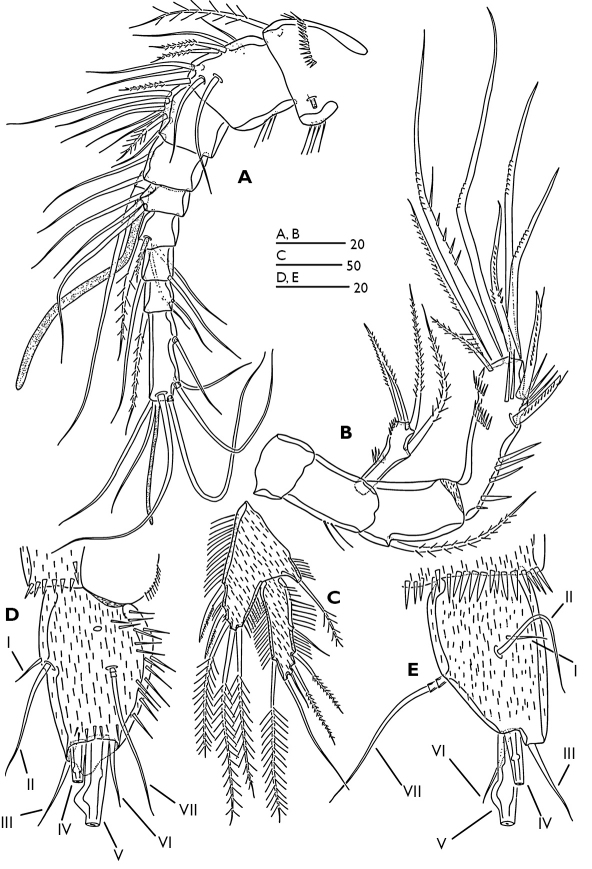
Odaginiceps korykosensis sp. n. Female. **A** antennule, dorsal **B** antenna **C** P5, anterior **D** left caudal ramus, dorsal **E** right caudal ramus, lateral.

Caudal rami (Figs 1C; 3D, E) tapering posteriorly with 4–5 dorsal spinules distally near the base of seta V; 1.7 times longer than wide; inner margin ornamented with spinules (Fig. 3D); with a pore on proximal third of dorsal surface (Fig. 3D), another pore present on ventral surface near the base of seta III (Fig. 2A); with seven setae (Fig. 3D, E), seta I minute located near the base of seta II; setae II–III bare; setae IV–V strongly developed and bipinnate; seta V with swollen sinuate base; seta VI short and as long as seta III; seta VII tri-articulated.

Antennule (Fig. 3A) 9-segmented; segment 1 with a long plumose seta at anterodistal margin, a spinular row on anterior surface, long spinules along inner margin, small tube-pore on dorsal surface near inner margin. Segment 2 with long spinules on caudal margin. Segment 4 with long aesthetasc fused basally to seta. Segment 9 longest, bears an apical acrothek consisting of a short aesthetasc and two setae. Armature formula 1-[1 plumose], 2-[6+3 plumose], 3-[6+1 plumose], 4-[3+(1+ae)], 5-[2], 6-[2+2 plumose], 7-[2], 8-[1+1 plumose], 9-[5+acrothek].

Antenna (Fig. 3B). Coxa small and smooth. Basis with 2 long spinules at outer margin. Exopod 1-segmented with one lateral pinnate seta, apical armature consists of one pinnate seta and one pinnate spine; a few spinules present around outer distal corner and midway along outer margin. Endopod 2-segmented; first endopod segment with one plumose seta at proximal third of outer margin. Distal endopod segment with various spinular rows as figured and with two abexopodal unipinnate spines laterally (both spines with subapical tubular extension). Apical armature of enp-2 consisting of two pinnate setae, and five geniculate setae; longest geniculate seta with large spinules and fused at base to long pinnate seta.

**Figure 4.**
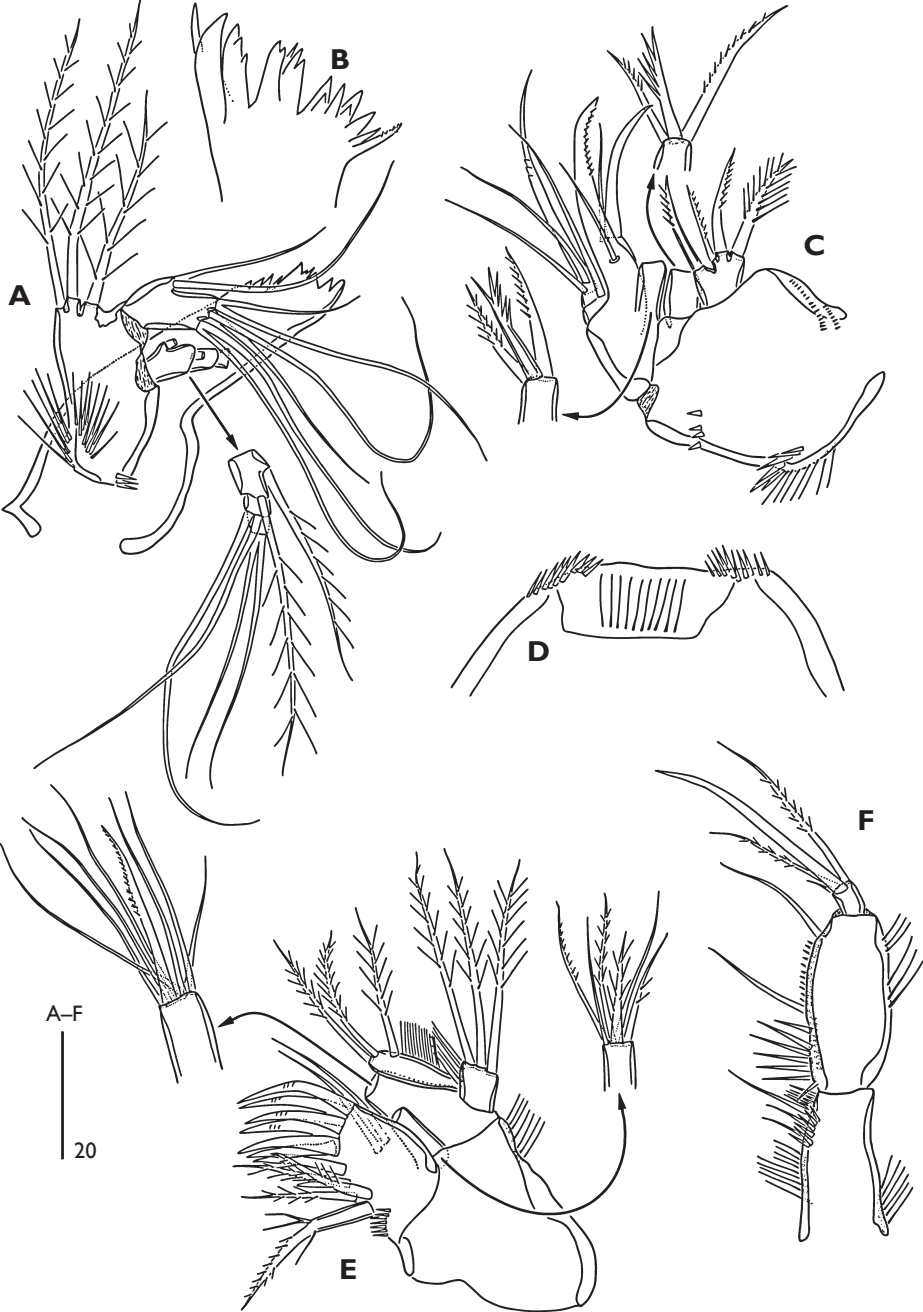
Odaginiceps korykosensis sp. n. Female. **A** mandible **B** distal margin of gnathobase **C** maxilla, posterior **D** labrum, ventral **E** maxillule, anterior **F** maxilliped, posterior.

Labrum (Fig. 4D). Free margin straight, with spinular row at distal corners and fine spinular row subdistally on ventral surface.

Mandible (Fig. 4A, B). Coxa robust, gnathobase with one pinnate seta at dorsal corner and several blunt multicuspidate teeth along distal margin. Palp biramous; basis strong, with three plumose setae along inner margin, ornamented with a group of long spinules proximally. Exopod 3-segmented, first segment with two plumose setae, second segment with two bare setae and third segment with two bare setae fused at base. Endopod 1-segmented with two lateral and six distal bare setae (two inner distal setae and two outer distal setae fused at base).

Maxillule (Fig. 4E). Praecoxal arthrite with seven spines around distal margin, with spinules as figured; anterior surface with two bare setae; posterior surface with three plumose and one pinnate setae. Coxal endite with two smooth and four plumose setae. Basis with seven bare and one unipinnate setae. Long endopod segment and square exopod segment each with three plumose setae. Endopod and exopod with a row of fine marginal setules.

Maxilla (Fig. 4C). Syncoxa ornamented with spinules as figured; with three endites. Proximal endite with one plumose and three unipinnate setae; middle endite with three unipinnate setae; distal endite with two unipinnate and one plumose setae. Allobasis drawn out into pinnate claw; accessory armature consisting of two bare setae and one curved spine. Endopod 2-segmented; proximal segment with one bare seta, distal segment with one geniculate and two bare setae.

Maxilliped (Fig. 4F). Subchelate and ornamented with spinular rows as figured. Syncoxa with three inner bare setae subdistally. Basis with two bare setae along inner margin. Endopod with one small accessory seta, one bare and two plumose setae.

**Figure 5.**
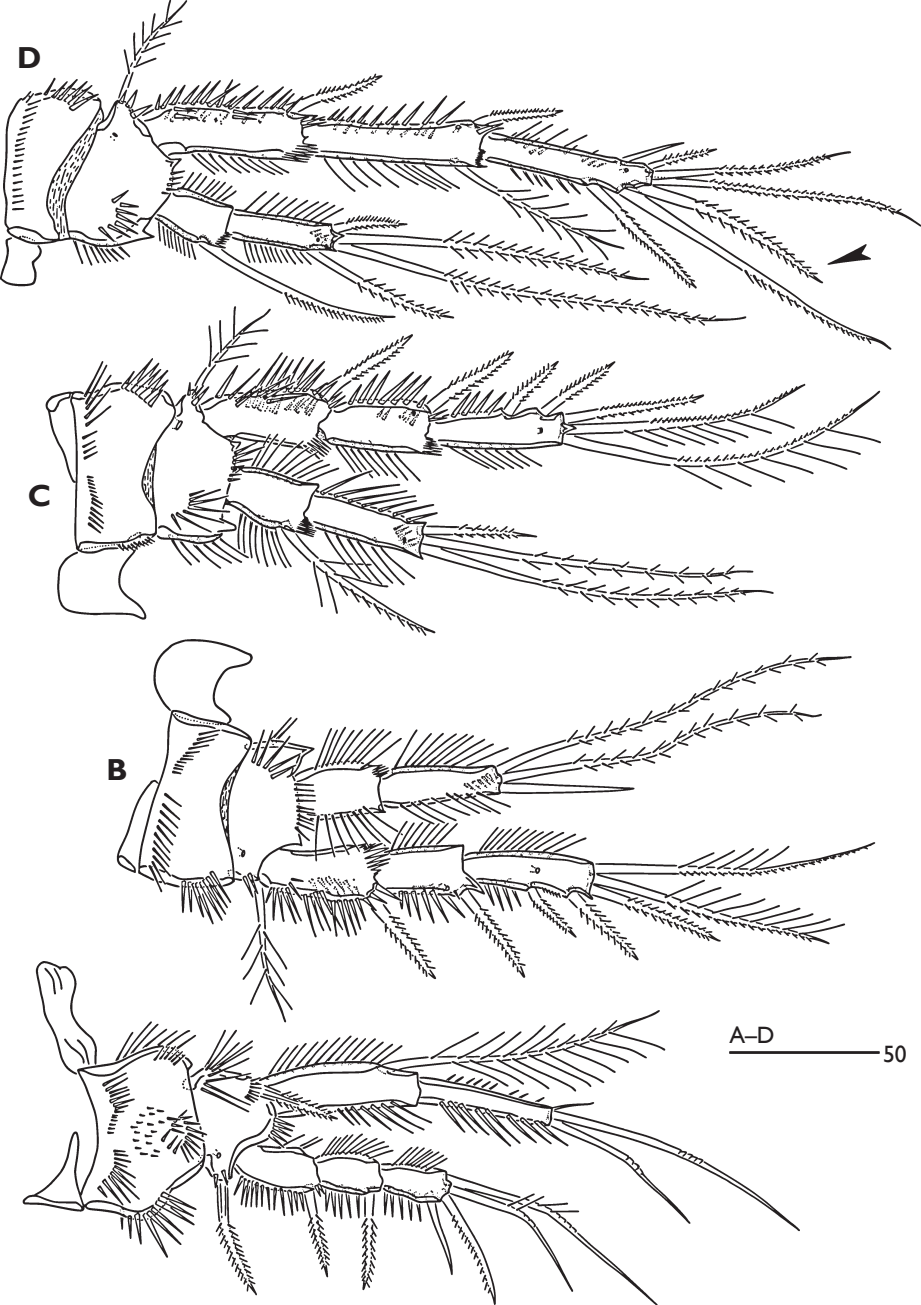
Odaginiceps korykosensis sp. n. Female. **A** P1, dorsal **B** P2, anterior **C** P3, anterior **D** P4, anterior.

P1 (Fig. 5A). Intercoxal sclerite rectangular and smooth. Small praecoxa triangular and bare. Coxa with complex spinular ornamentation anteriorly as figured. Basis narrower than coxa; anterior surface with pore near the base of outer bipinnate spine; inner side with long slender spinules. Exopod 3-segmented, segments with spinular rows along inner and outer margins. Endopod 2-segmented; enp-1 reaching almost middle of exp-3, with spinular row along inner and outer margins; long inner seta plumose and located subdistally; enp-2 slightly shorter than enp-1, with spinules along inner and outer margins and with two long articulated setae and one small inner apical seta.

P2–P4 (Fig. 5B–D). Intercoxal sclerite unornamented. Coxa and basis with complex spinular ornamentation as figured. Exopod 3-segmented. Endopod 2-segmented. Endopodal and exopodal segments with spinular rows along inner and outer margins. Exp-1 of P2-P3 without spinules along inner margin. Enp-1 of P2 without inner seta. Terminal outer seta of P2 enp-2 bare. P4 exp-2 with one plumose inner seta. With a pore on anterior surface of enp-2 and anterior surface of exp-2 and -3. Exp-1 and -2 (and -3 in P4) with a posterior spinule patches. Enp-2 with a posterior spinular row subdistally.

Armature formula of swimming legs:

**Table:** 

P1		P2		P3		P4	
Exp.	Enp.	Exp.	Enp.	Exp.	Enp.	Exp.	Enp.
0.0.022	0.120	0.0.023	0.021	0.0.023	1.021	0.1.322 (♀)	1.121
						0.1.222 (♂)	

P5 (Fig. 3C). Baseoendopod and exopod covered with fine spinules on anterior surface, with long slender spinules along inner and outer margins. Exopod 3.6 times longer than wide, with 6 setae; seta 1 longest and plumose; seta 3 smallest and bare. Baseoendopod longer than wide; with 2 unipinnate and 3 plumose setae.

P6 (Fig. 2A) represented by a small segment with one outer plumose seta and two slender bare setae.

#### Description.

**Figure 6.**
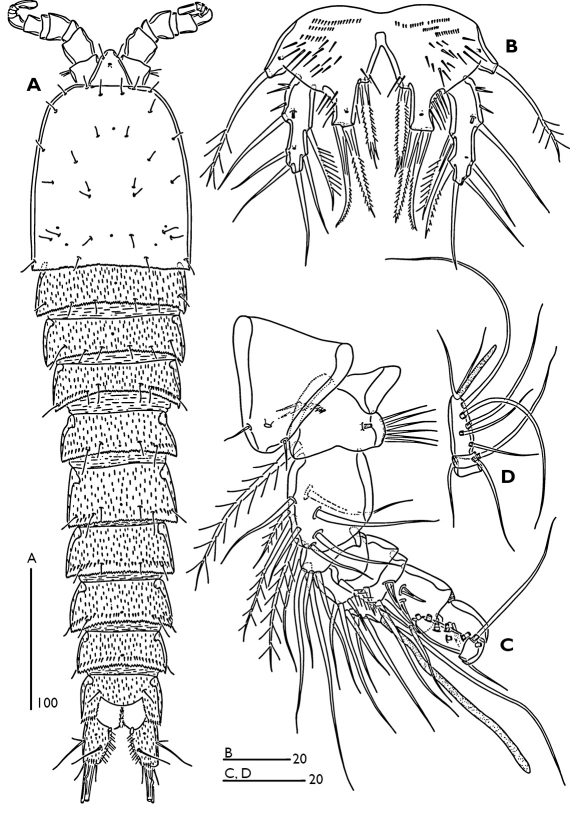
Odaginiceps korykosensis sp. n. Male. **A** habitus, dorsal **B** P5, anterior **C** rostrum and antennule, dorsal **D** terminal segments of antennule.

Male (Fig. 6A). Total body length 510 µm. Body smaller and more slender than female, largest width measured at midway of cephalothorax. Body ornamentation generally as in female. Sexual dimorphism observed in antennule, P2-P6 and genital segmentation.

Antennule (Fig. 6C, D) indistinctly 10-segmented, sub-chirocer. Segment 1 short, with small tube-pore on dorsal surface and with long spinules along caudal margin. Segment 2 longest. Segment 4 with partial suture line dorsally. Segment 5 with long aesthetasc fused basally to seta. Segment 10 bears an apical acrothek consisting of a short aesthetasc and two slender setae. Armature formula 1-[1 plumose], 2-[7+3 plumose], 3-[4], 4-[4+1 plumose], 5-[4+1 spine+(1+ae)], 6-[1+2 spines], 7-[2], 8-[1], 9-[2], 10-[5+acrothek].

P2 enp-2 (Fig. 1D); outer terminal spine more robust than female; middle terminal seta bare, shorter than female and as long as outer spine; inner terminal seta minute. P3 enp-2 (Fig. 1E); inner terminal seta modified to a short spine (arrowed in fig. 1E). P4 exp-3 (Fig. 1F) with 6 setae, inner terminal seta of female (arrowed in fig. 5D) absent in the male.

P5 biramous (Fig. 6B), fused medially. Baseoendopod ornamented with patch of spinules as figured; with two pores (one near the base of outer basal seta and the other near the base of inner terminal spine of endopodal lobe); endopodal lobe with one lateral and two distal spines. Exopod with three outer bare setae, 1 terminal bare seta and two inner unipinnate setae; with two pores (one tube pore near the base of outer proximal bare seta and the other near the base of outer median bare seta). P6 vestiges asymmetrical (Fig. 2B); each P6 with one plumose inner seta and two long bare setae.

#### Etymology.

The specific name refers to “korykos” which is the historical name of Kızkalesi province (Mersin, TURKEY).

## Discussion

The new species can be attributable to the genus Odaginiceps by the absence of inner seta on the proximal segments of P2-P4 exopod and P2 endopod, the short second antennulary segment, the large prominent rostrum, the presence of pinnate setae on the second and third antennulary segments, and the two-segmented P1 endopod with three armature elements on the second segment (Fiers 1995; Fiers and De Troch 2000). Thus far, four species have been assigned to the genus Odaginiceps: Odaginiceps clarkae, Odaginiceps xamaneki, Odaginiceps elegantissima and Odaginiceps immanis. The new species can easily be distinguished from its congeners by the presence of four setae/spines on the second endopodal segment of P4, by the shape of the caudal rami and by the occurrence of four setae on the proximal endite of maxilla. The new species is most closely related to the Odaginiceps xamaneki. Both species differ from the 3 other congenerics by the presence of short, and compact caudal rami (long and semi-cylindrical in the other species) and the spinular posterodorsal ornamentation of the urosomites (spinular rows present only ventrally in the 3 other species). Odaginiceps korykosensis sp. n. (female) differs from Odaginiceps xamaneki by the presence of spinules (interrupted midway) along the posterioventral margin of genital double-somite, by the absence of a central spinular patch on ventral surface of second abdominal somite, by the longer outermost seta of P5 baseoendopod, by the much longer terminal plumose setae of P2-P3 enp-2, by the longer P3 enp-2, as well as other minor differences observed on the spinular ornamentations of various appendages.

Fiers (1995) assumed that the presence of an inner pectinate element on the P3 enp-1, the presence of an inner seta on P4 exp-2, and the distinctly shorter caudal rami in both sexes could be sufficient grounds to erect a new genus to accommodate Odaginiceps xamaneki. The above mentioned potential generic characters for Odaginiceps xamaneki are also observed in Odaginiceps korykosensis which supports the previously formulated assumption. However, until the specific and generic importance of these characteristics are better understood and evaluated, both species are retained in the genus Odaginiceps.

The new species lacks a seta (arrowed in the Fig. 5D) on the third exopodal segment of the P4 in the male. The absence of this seta in the male supports the assumption of Fiers (1995) that almost all tetragonicipitid males bear one seta less on the third exopodal segment of the P4.

Note on the ecology and distribution. Examination of the extensive interstitial samples taken from almost all sandy beaches along the Turkish coasts revealed that the new species occurs at the type locality only. On the other hand three samples taken from the type locality in different seasons revealed no true interstitial forms. The absence of true interstitial forms but the presence of Odaginiceps korykosensis sp. n. in the type locality can be explained by the very low oxygen levels measured at the type locality (Table 1). Most harpacticoids are sensitive to reduced oxygen supply, which restricts their occurrence to the upper sediment layers and favours epibenthic life. It can be discerned from the general body shape of Odaginiceps spp. (see Fig. 1A, B; Fiers 1995; Fiers and De Troch 2000) that they are not truly interstitial, but are probably epibenthic forms crawling on/in the upper surface of sediment along the shallow/deeper waters, meaning that two specimens of Odaginiceps korykosensis sp. n.accidentally entered into the interstitial sample. The effect of the oxygen supply on the horizontal composition of harpacticoid species in the sediment can be supported by the presence of several interstitial forms (such as Arenosetella Wilson, 1932) found in 95 % of stations sampled along the Mediterranean coast of Turkey (unpublished data). The oxygen levels in the sediments in these localities were much higher than those observed at the locality of Odaginiceps korykosensis sp. n.

## Supplementary Material

XML Treatment for Odaginiceps korykosensis
